# Street Cleaning in Large Cities

**Published:** 1891-04

**Authors:** 


Street Cleaning in Large Cities.—This subject is discussed in the April number of the *Popular Science Monthly* by Col. Emmons Clark, of the famous New York "Seventh." The paper includes explicit practical suggestions for the proper performance of this important work, and it might be advisable for the Buffalo Common Council and the Street Commissioner's Bureau to subscribe for a few copies.

---

Dr. Joseph Price, of Philadelphia, has recently completed a series of 900 abdominal sections, embracing every variety of disease and condition for which the abdomen properly may be opened, with twenty-seven deaths—a mortality of precisely three per cent. for the whole series. These sections embrace thirty-eight consecutive successful abdominal hysterectomies, and these latter include three

Porro-Cesarian sections. In the series are included fifty-four extra-uterine pregnancy operations, with two deaths.

This record readily gives Dr. Price the distinction of being the foremost American abdominal surgeon, and in some respects it has not been equaled in the world.

